# Applications of Light-Sheet Microscopy in Microdevices

**DOI:** 10.3389/fnana.2019.00001

**Published:** 2019-01-25

**Authors:** Ignacio Albert-Smet, Asier Marcos-Vidal, Juan José Vaquero, Manuel Desco, Arrate Muñoz-Barrutia, Jorge Ripoll

**Affiliations:** ^1^Department of Bioengineering and Aerospace Engineering, Universidad Carlos III de Madrid, Madrid, Spain; ^2^Experimental Medicine and Surgery Unit, Instituto de Investigación Sanitaria del Hospital Gregorio Marañón, Madrid, Spain; ^3^Centro de Investigación Biomédica en Red de Salud Mental (CIBERSAM), Madrid, Spain; ^4^Centro Nacional de Investigaciones Cardiovasculares Carlos III (CNIC), Madrid, Spain

**Keywords:** light-sheet fluorescence microscopy, SPIM, microdevices, microfluidics, cellular imaging

## Abstract

Light-sheet fluorescence microscopy (LSFM) has been present in cell biology laboratories for quite some time, mainly as custom-made systems, with imaging applications ranging from single cells (in the micrometer scale) to small organisms (in the millimeter scale). Such microscopes distinguish themselves for having very low phototoxicity levels and high spatial and temporal resolution, properties that make them ideal for a large range of applications. These include the study of cellular dynamics, in particular cellular motion which is essential to processes such as tumor metastasis and tissue development. Experimental setups make extensive use of microdevices (bioMEMS) that provide better control over the substrate environment than traditional cell culture experiments. For example, to mimic *in vivo* conditions, experiment biochemical dynamics, and trap, move or count cells. Microdevices provide a higher degree of empirical complexity but, so far, most have been designed to be imaged through wide-field or confocal microscopes. Nonetheless, the properties of LSFM render it ideal for 3D characterization of active cells. When working with microdevices, confocal microscopy is more widespread than LSFM even though it suffers from higher phototoxicity and slower acquisition speeds. It is sometimes possible to illuminate with a light-sheet microdevices designed for confocal microscopes. However, these bioMEMS must be redesigned to exploit the full potential of LSFM and image more frequently on a wider scale phenomena such as motion, traction, differentiation, and diffusion of molecules. The use of microdevices for LSFM has extended beyond cell tracking studies into experiments regarding cytometry, spheroid cultures and lab-on-a-chip automation. Due to light-sheet microscopy being in its early stages, a setup of these characteristics demands some degree of optical expertise; and designing three-dimensional microdevices requires facilities, ingenuity, and experience in microfabrication. In this paper, we explore different approaches where light-sheet microscopy can achieve single-cell and subcellular resolution within microdevices, and provide a few pointers on how these experiments may be improved.

## Introduction

Research in the area of cell motility (Ridley, 2003; Mayor and Etienne-Manneville, 2016) is essential to understand many core life processes like development and neurology, with especially strong biomedical significance in the fields of immunology, tissue repair, and tumor metastasis. To study these dynamic processes, imaging technologies that can achieve fast temporal resolution and subcellular spatial resolution are required. Light-sheet microscopy can offer the spatio-temporal resolution necessary to record many of these phenomena, while microdevices have proven themselves a useful tool in cell culture and motility experiments. For imaging in microdevices, a variety of optical methods are available (see Wu et al., 2012 for a review on this subject). Our review will focus on emerging and established methods where cell-resolution light-sheet techniques can be combined with bioMEMS (bio-microelectromechanical systems) to exploit the advantages of both technologies.

The structure of this paper consists of four sections. Section “Introduction” will serve as an introduction and is devoted to explaining the basics of light-sheet microscopy and microdevices. In Section “Light-Sheet Optical Systems for Standard Sample Mounting,” general light-sheet configurations for standard sample mounting are described. Section “Integrated Light-Sheet Microscopy and Microdevices” discusses a variety of approaches that have integrated light-sheet illumination and microdevice technology. Finally, an outlook is drawn is Section “Discussion and Outlook,” where a few pointers on how these experiments could be prospectively improved are discussed.

### Light-Sheet Fluorescence Microscopy

#### Introduction to Fluorescence Light-Sheet Microscopy

Light-Sheet Microscopy or Ultramicroscopy, originally developed in 1903 (Siedentopf and Zsigmondy, 1903), became an emerging technology in 2004 when it was applied for the first time to *in vivo* imaging (Huisken et al., 2004) after initial description of the technique for imaging cleared samples (Voie et al., 1993; Santi et al., 2009). Light-sheet microscopy is based on generating a sheet of light within the specimen and ensuring it coincides with the focal plane of a high numerical aperture objective placed at 90°. How one generates this light-sheet will determine how sensitive the setup is to scattering. The original setups make use of a cylindrical lens to generate the light-sheet. This approach is named selective plane illumination microscopy (SPIM) and can be combined with multi-photon excitation to improve spatial resolution and penetration even further (Planchon et al., 2011; Gao et al., 2012). Resolution improvement may also be achieved through engineering complex light-sheets and coupling these with the detection scheme as in Lattice Light-Sheet microscopy (Chen et al., 2014), a technique useful for imaging small volumes *in vivo*. Note that even though planes of light can be created directly from Gaussian beams using cylindrical lenses, beams of other shapes can be digitally scanned to form virtual light-sheets (Keller et al., 2008; Chen et al., 2014) by a technique known as digitally-scanned lightsheet microscopy (DSLM). The optical aspects of a light-sheet microscope have been discussed in more detail by Weber et al. (2014) and Olarte et al. (2018).

Nevertheless, fluorescence light-sheet microscopy is not the only optical sectioning technique available. How light-sheet fluorescence microscopy (LSFM) compares to other fluorescence microscopy techniques has been previously reviewed (Fischer et al., 2011; Ripoll et al., 2015; Combs and Shroff, 2017). Most often, comparisons are drawn between light-sheet microscopy and confocal fluorescence microscopy, with the latter being the most widespread sectioning technique in biological laboratories. There are different types of confocal techniques (Jonkman and Brown, 2015), the two prominent being confocal laser-scanning microscope (CLSM) and spinning-disk confocal microscope (SDCM). The strengths LSFM can offer are higher speed, lower phototoxicity and higher penetration compared to confocal microscopy, as long as the imaging geometry allows it. Note that, if several wavelengths need to be imaged simultaneously, LFSM requires splitting the complete image by means of dichroic mirrors. Thus, the number of simultaneous wavelengths that may be imaged is limited, typically imaging two or three bands at most. On the contrary, confocal methods offer higher resolution, are capable of imaging many emission wavelengths simultaneously, and make use of a single objective for illumination and detection.

#### Applications of Light-Sheet Microscopy

There are a wide range of imaging applications that exploit the advantages of LSFM to perform fast volumetric scans of biological samples and can be broadly classified by the size of the specimen being imaged (Power and Huisken, 2017). With regards to neurological morphology and activity studies (Gualda et al., 2014; Keller and Ahrens, 2015; Haslehurst et al., 2018), these have been imaged at different scales: from nervous systems of small organisms to single neurons.

Scanning of large specimens relies on the penetration of light through tissues which is limited in confocal microscopy due to the illumination and detection geometry. Studies of this kind include developmental biology in small organisms, mostly in embryos and larvae (Huisken and Stainier, 2009; Ripoll et al., 2015), and cleared organs (Keller and Dodt, 2012; Arranz et al., 2013; Nehrhoff et al., 2016, 2017; Ozga et al., 2016; Susaki and Ueda, 2016; Gómez-Gaviro et al., 2017). It is in this area where the first biological application of orthogonal light-sheet illumination – named orthogonal-plane fluorescence optical sectioning (OPFOS) – was applied to image the inner ear cochlea of a guinea pig (Voie et al., 1993).

At smaller scales, LSFM has also been used in imaging spheroid cultures and single cells (Verveer et al., 2007; Lorenzo et al., 2011; Pampaloni et al., 2013; Liu et al., 2015). A growing importance of spheroid and 3D cultures (Edmondson et al., 2014; Ancora et al., 2017) requires higher imaging depth, an advantage offered by LSFM.

For cell-sized samples, even though other fluorescence methods may offer higher spatial resolution, it is the temporal resolution and low photodamage of LSFM that is attractive. Fast imaging becomes particularly important in dynamic processes such as cell motion (migration and development) and tracking of vesicles, cell lineage differentiation, and recording of Ca^2+^ activity. When sequential volumetric images of these phenomena are desired, most imaging techniques are limited in sampling frequency. Here, LSFM offers the highest temporal resolution of all sectioning techniques. In this context, it is worth mentioning that a particular light-sheet technique, namely Lattice light-sheet microscopy (Chen et al., 2014), has achieved remarkable spatiotemporal resolution in multiscale applications, including single molecules, cell spheroids, mitotic microtubule dynamics, immunological synapse, cell motility in a 3D matrix, and embryogenesis in small organisms’ embryos. Lattice LSFM microscopy can be implemented in some of the setups described in Sections “Light-Sheet Optical Systems for Standard Sample Mounting” and “Integrated Light-Sheet Microscopy and Microdevices” as shown in Table 1.

**Table 1:** Light-sheet fluorescent microscopy techniques that can provide imaging in microdevices.

Technique	# Objective	Objective-sample orientation	Resolution isotropy	Sectioning method	Relative cost	Sample static	Lattice light-sheet
SPIM	2	0 and 90°	2D	Mechanical or ETL	Reference value, N/A	No^∗^	Yes
Tilted LSFM	2	0 and <90°	2D	Mechanical	Comparable	No	No
Oblique plane microscopy (OPM)	1	0°	Non-linear 2D	Mechanical (SCAPE)	Comparable	No	No
Inverted SPIM (iSPIM)	2	-45 and +45°	2D	Mechanical or ETL^∗^	Higher	Yes	Yes
Dual-illumination iSPIM (diSPIM)	2	-45 and +45°	3D	Mechanical or ETL^∗^	Higher	Yes	Yes
Open-top SPIM	2	-135 and +135°	2D	Mechanical or ETL^∗^	Higher	No^∗^	Yes
Diagonally-scanned SPIM	2	-45 and +45°	2D	Mechanical or ETL^∗^	Comparable	No^∗^	Yes
Single-objective SPIM (soSPIM)	1	0°	2D	Mechanical or ETL^∗^	Comparable	Yes	No
Reflected LSFM (RSLM)	2	0 and 180°	2D	Mechanical or ETL^∗^	Comparable	No^∗^	No

**Figure 1:**
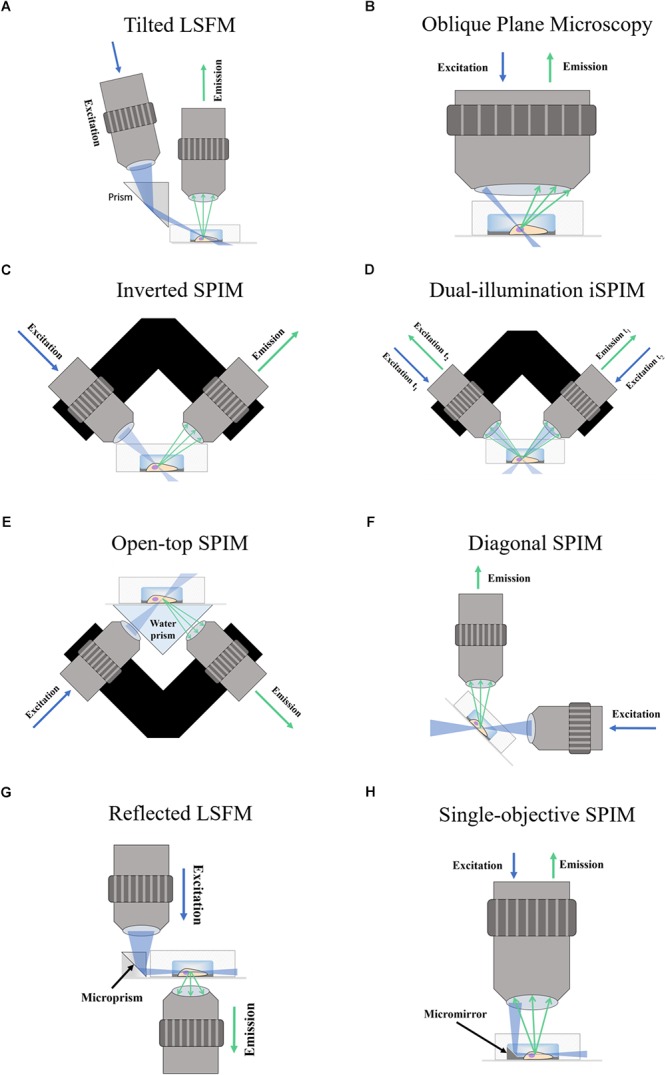
Schematic of Fluorescence Light-Sheet Microscopy techniques. The figure shows illustrations of the different LSFM techniques for imaging in microdevices. **(A)** Titled LSFM; **(B)** Oblique Plane Microscopy; **(C)** Inverted SPIM; **(D)** Dual-illumination iSPIM; **(E)** Open-top SPIM; **(F)** Diagonal SPIM; **(G)** Reflected SPIM; **(H)** Single-objective SPIM. In the diagrams, the blue arrows denote the direction of excitation light traveling the objective, while the green arrows show the path of emission light. The light-sheet created is shown in light blue, and focuses in the specimen (a cell) within a microfluidic device supported on a coverslip. In Oblique Plane Microscopy **(B)** the size of the objective is disproportionally large to represent that an objective of high numerical aperture is required.

*The table summarizes and compares the main characteristics of the LSFM optical setups that can perform scans in microdevices. The labels in each column are described next. *Non-linear resolution*: in Oblique Plane Microscopy the resolution in every direction is a function of the angle of the oblique illumination plane. This also applies to Tilted LSFM; however, in this approach, the angle does not change during the scan. *Mechanical*: sectioning performed by a piezoelectric stage, fluid flow, or other mechanically-driven method. *SCAPE* (Bouchard et al., 2015) is a unique Oblique Plane Microscope scanning method. *ETL*: an electrically tunable lens drives sectioning. *ETL^∗^*: a mirror galvanometer and an electrically tunable lens should be able to drive sectioning, but there is no published material. The column “Objective-Sample Orientation” describes the angle at which the objective(s) is placed with respect to the sample. An angle of 0° is equivalent to an upright microscope orientation, and 180° to that of an inverted microscope. For diagrams illustrating this please refer to Figure 1. The column “Relative Cost” compares the cost of hardware compared to a traditional SPIM (Note: SCAPE is not accounted for in this comparison). The column “Sample Static” states whether or not the sample remains static during the scan. The label *No^∗^* means that it should possible if ETL scan is implemented, but there is no published material. The column “Lattice Light-sheet” states whether or not lattice LSFM could be compatible with that setup*.

A number of setups and applications offering cellular resolution are described in Sections “Light-Sheet Optical Systems for Standard Sample Mounting” and “Integrated Light-Sheet Microscopy and Microdevices,” as well as flow cytometry and single-molecule detection arrangements. This review will focus on single-cell and subcellular resolution setups, more specifically those that have accomplished or can accomplish imaging within microfluidic devices.

### Microdevices

Microdevices, also referred to as bioMEMS (bio-microelectromechanical systems), are miniaturized devices with electrical and/or mechanical properties that are used in biological applications. These include a diversity of microfabricated tools from sensors and actuators (atomic force microscopy cantilevers and micropumps) to microfluidic devices (Huang, 2013). Most microdevices covered here will be microfluidic devices (Whitesides, 2006; Sackmann et al., 2014), which have become popular in cell biology laboratories as an instrument for precise sample manipulation and culturing. The progress made in the field of microfluidic devices to replace traditional experimental approaches in diagnostics and cell biology has been covered elsewhere (Lindström and Andersson-Svahn, 2010; Huang, 2013; Sackmann et al., 2014).

Microfluidic devices can be broadly categorized into three groups. In the first group the aim is toward high-throughput and automation and relies on complex and highly-controlled devices with integrated pumps and valves. These systems may require a high development and setup investment, but result in large reductions in labor and reagent cost. Machines for automated drug screening, cell culturing and monitoring, and some high-end point-of-care diagnostic units would fall into this category. Another group consists on finely-tuned devices for low-scale experiments: 3D *in vitro* cultures, drug screening and organ-on-a-chip. The goal here is to create the desired conditions in a reproducible way to investigate a certain biological phenomenon with high control and high fidelity to produce relevant results. These bioMEMS are not necessarily costly in terms of hardware but require a certain degree of technical expertise to use and prepare. Finally, the third category encompasses low-cost and simple systems that can be easily fabricated and utilized without advanced engineering facilities or expert knowledge. Generally, these inexpensive devices are disposable to avoid contamination. These aim to reduce the costs of experimentation and diagnostics in biology or medicine to make it more accessible or marketable.

The microdevices covered in this review are those that have been used successfully with light-sheet illumination to perform more advanced biological experiments regarding imaging speed, quality and/or throughput.

## Light-Sheet Optical Systems for Standard Sample Mounting

As previously mentioned, fluorescence light-sheet microscopy has demonstrated great advantages in terms of speed and low phototoxicity at several levels of resolution. However, there are some limiting aspects that have stalled its presence in cell biology laboratories. For example, initial designs of LSFM systems would require the specimen to be embedded in an agarose gel or inside a Fluorinated Ethylene Propylene (FEP) tube (Kaufmann et al., 2012), which would rotate around its axis to record multiple views, and was translated linearly through the plane of light to acquire sections (Huisken et al., 2004; Preibisch et al., 2010; Mickoleit et al., 2014). The views and slices acquired were later fused into the final 3D image. This proved to be inconvenient for *in vitro* cell imaging where the sample is typically placed on static horizontal surfaces. Furthermore, spinning-disk confocal microscopy had already enabled fast imaging (10s of frames per second) with confocal setups for single-cell or thin-culture applications where high penetration is not generally an issue. An additional advantage of confocal microscopy is that, similarly to other microscopes of widespread use (e.g., phase contrast, brightfield, and widefield), it only requires one optical axis for illumination and detection making it very convenient for standard sample mounting. On the other hand, light-sheet systems generally require two axes placed at 90° from each other, demanding a new way of mounting the sample.

As an emerging field of optical hardware, light-sheet microscopy is dominated by custom-made setups. There are a number of open access publications (Gualda et al., 2013, 2015; Pitrone et al., 2013; Gao et al., 2014; Olarte et al., 2018) and guidelines that explain how to build different light-sheet configurations, several of which are included in this review. Although the diversity of customized systems has offered higher control over image acquisition, many require a certain degree of optical expertise that may not be available to many experimental biology groups. In this context, it has been troublesome to keep track of the light-sheet configurations available and their features. Many LSFM research groups have coined a variety of names and acronyms for configurations that are very similar in the eyes of those who may not be familiarized with optics but are potential users of such equipment. In the next subsections, the main light-sheet setups that could be applied to cell imaging and their characteristics are described. An overview is shown in Table 1.

### Tilted Light-Sheet Microscopy

In tilted light-sheet methods the illumination plane is not completely parallel to the detection objective which allows the sample to be accessed sideways from the top. In the setup proposed by Fadero et al. (2018), a photomask is used to create a light-sheet with a wedge-like shape which converges when parallel to the objective. Gustavsson et al. (2018) make use of a glass prism next to the sample to create a plane of light incident at an angle (Figure 1A) and validated this approach by imaging mitochondria and the nuclear lamina. Both setups can be built within a standard microscope and employ piezoelectric motors for volumetric scanning.

### Oblique Plane Microscopy

Oblique plane microscopy (Dunsby, 2008), or OPM, employs a single objective of high numerical aperture for both illumination and detection at an angle (Figure 1B). A tilting mirror is used to select the angle at which the light-sheet enters the sample through the objective. The “tilted” fluorescence image captured is then refocused into a digital camera and a motorized stage is proposed for volumetric scanning. The possibility of imaging microfluidic devices for lab-on-a-chip, cytometry and other applications is also discussed. An adapter designed by Cutrale and Gratton (2012) could facilitate its integration into standard microscope setups for commercialization. Oblique plane microscopy has been successfully implemented in single-cell imaging of yeast cells in coverslips (Theer et al., 2016).

The most exciting example of OPM is swept, confocally-aligned planar excitation (SCAPE) microscopy (Bouchard et al., 2015), which acquires full volumes of living samples at speeds in the order of 20 volumes per second. This is done by confocal de-scanning and image rotation optics that map an oblique moving plane onto a stationary high-speed camera while the sample remains static. SCAPE microscopy has been successfully applied to spontaneous neuronal firing in the intact brain of living mice and live *Drosophila* larvae. The greatest advantage of OPM is the use of a single objective; other techniques that require two-objectives (one for illumination and one for detection) pose restrictions regarding sample geometry due to the different optical paths.

### Inverted SPIM Configurations

There are several optical set-ups that would fall into the wider category of inverted SPIM (iSPIM), a simple arrangement to facilitate imaging in standard-mounted samples such as coverslips, slides, wells and polydimethylsiloxane (PDMS) microdevices. iSPIM includes some distinct redesigned versions such as dual-illumination iSPIM (diSPIM), open-top SPIM, and diagonally-scanned SPIM which will be discussed in this section.

#### Inverted SPIM

The inverted SPIM setup (Wu et al., 2011), or iSPIM, was intended to resolve one of the main issues of LSFM for standard sample mounting which requires two optical paths to access the specimen for illumination an detection. In iSPIM, emission and excitation occur at 90° of each other and at 45° from the horizontal surface where the sample is placed (Figure 1C). A motorized stage translates the optics vertically to acquire volume scans. Inverted SPIM setups have successfully been used to image neurons (Holekamp et al., 2008) very early on since SPIM emerged. Studies in the field of neuroscience have greatly benefitted from the speed of LSFM, which includes neurodevelopmental dynamics in tracking axon growth and neuron migration in *C. elegans* (Wu et al., 2011), neural calcium imaging (Yang et al., 2016; Haslehurst et al., 2018) and subcellular calcium puffs (Ellefsen and Parker, 2018). The latter reported that, although conventional LSFM is the fastest technique, it could only achieve dynamic calcium imaging when imaging a single slice.

**Figure 2:**
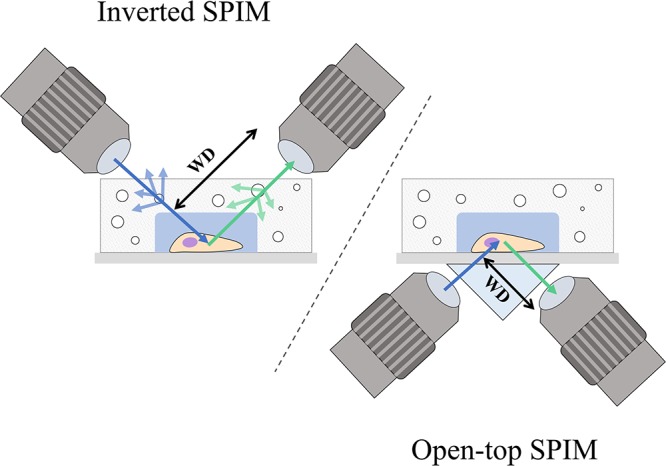
Comparison between the inverted SPIM and open-top SPIM configurations. Illustrations of iSPIM and open-top SPIM are shown imaging a cell in a PDMS microfluidic device with some bubbles. The blue arrows denote excitation light, while the green arrows show the path of emission light. In iSPIM, lighter blue and lighter green arrows show the reflectance caused by bubbles, which is avoided in open-top SPIM by imaging through the refractive medium prism and the glass coverslip. The working distance (WD) of both setups is illustrated, exemplifying another of the advantages of open-top SPIM which allows objectives of shorted working distance which are generally those of higher numerical aperture and magnification.

A possible drawback of applying inverted SPIM to image PDMS microfluidic devices or wells could arise from the working distance of the objectives (see Figure 2). The choice of objectives could be limited by the height of the well or the PDMS layer, especially among those of high numerical aperture and high magnification which typically have shorter working distances and are fundamental for subcellular resolution imaging. As discussed later in Section “Inverted SPIM Configurations,” this could be ameliorated with an open-top SPIM configuration.

#### Dual-Illumination Inverted SPIM

Dual-illumination iSPIM (Wu Y. et al., 2013; Kumar et al., 2014), or diSPIM, extends iSPIM by making use of both orthogonal objectives to sequentially acquire two views of an image (Figure 1D) which are merged during post-processing to produce a dataset of 3D isotropic resolution. Using this approach, and in general any approach which involves combining several views (Preibisch et al., 2010; Krzic et al., 2012; de Medeiros et al., 2015), achieves a reduction in shadow artifacts caused by the generation of unwanted stripes due to the presence of strongly absorbing or scattering objects (Huisken and Stainier, 2007, 2009). This additional feature further improves the image quality in diSPIM.

The main drawbacks to diSPIM with respect to iSPIM are an increase in photobleaching and a decrease in time resolution because two full images – one from each objective – must be acquired. Recall that temporal resolution and low phototoxicity are two core advantages of LSFM with respect to other optical sectioning techniques. Additionally, post-processing may take several hours for large data sets, while regular LSFM systems acquire directly the final image and do not require post-processing. Still, when comparing diSPIM to spinning disk microscopy, diSPIM demonstrated threefold faster sampling and exhibited significantly lower bleaching than spinning disk (Wu Y. et al., 2013). Also, if required by an experiment, dual-illumination iSPIM systems can be operated like a regular iSPIM for faster speed and lower bleaching. Note that diSPIM setups require two or three high-speed high-resolution cameras.

The same group has developed an interesting reflective diSPIM method (Wu et al., 2017) that employs commercially available reflective coverslips to provide additional views, which are fused by computationally-intensive post-processing. The illumination plane reflects off the coverslip creating a second orthogonal light-sheet that allows for simultaneous rather than sequential diSPIM imaging. Regarding emission, the fluorescence light that would be lost through the coverslip reflects off the coverslip and can be detected, creating a virtual mirror image on the underside of the coverslip. Compared to diSPIM, reflected diSPIM allows for volume images of even higher spatiotemporal resolution to be acquired for studying mitochondrial, membrane, Golgi, and microtubule dynamics in cells and calcium activity in nematode embryos.

#### Open-**Top** SPIM

Open-top SPIM (McGorty et al., 2015) follows a similar setup to iSPIM but instead of having the objectives above the sample these are located underneath (Figure 1E). The objectives conveniently face a “refractive prism” that can accommodate media of different refractive indices, thus alleviating the effects of refraction and distortion of the light-sheet. Imaging from underneath can mitigate the working-distance problem of the iSPIM setup, which can be especially relevant when imaging PDMS microfluidic devices. These devices are generally created with a thicker layer of PDMS above the channels, which are sealed from underneath by a slide or coverslip. The problem of bubbles typically present in the PDMS that could interfere with the illumination and detection paths (creating the above-mentioned shadow artifacts) is also tackled when imaging through the glass coverslip instead of through the PDMS gel (Figure 2). It should also be possible to design a dual-illumination open-top SPIM for isotropic spatial resolution and reduced shadow artifacts from merging different views.

In their study, McGorty et al. (2015) demonstrate the possibility of imaging GFP-expressing central nervous system neurons in *Drosophila* developing embryos within a PDMS microfluidic device under highly controlled conditions. In a posterior study (Mcgorty et al., 2017), the authors were able to image the nuclear lamina of fluorescently-labeled human embryonic cells and introduce some post-processing software for aberration correction. The main drawback of this approach is that a mechanical stage translates the sample during the scan. Inertial forces caused by vibrations and movement of the motorized stage could negatively affect the finely-tuned conditions within a microdevice. Alternatively, slow and precise translation stages can be used, but this leads to a significant decrease in sectioning speed.

#### Diagonally-Scanned SPIM

Diagonally-scanned SPIM (Dean et al., 2016) is similar to inverted SPIM, but instead of turning the optics 45° for the sample to remain horizontal, it is the optical system that stays horizontal and the sample is mounted at an inclined plane (Figure 1F). As a custom-made system, it should be possible to arrange it with an inverted SPIM configuration if desired. An interesting element of this setup (Dean et al., 2016) is that an accelerometer is used to analyze the effects of vibration and movement in image quality, showing that collagen hydrogels mimicking extracellular matrices (ECM) suffer from degraded image quality when rapidly scanning the sample. The diagonally-scanned light-sheet microscope was applied to 3D live-cell imaging in 2D substrates of microtubule and actin cytoskeletal dynamics, phosphoinositide signaling, clathrin-mediated endocytosis, polarized blebbing, and endocytic vesicle sorting, not all of which were successfully accomplished due to sub-optimal temporal resolution. The authors suggest the use of electrically tunable lenses (ETLs) (see Fahrbach et al., 2013, for example) as an alternative scanning method to mechanical translation to achieve the required imaging speeds.

Both diagonally-scanned SPIM and an earlier publication by this group on axially swept light-sheet microscopy (Dean et al., 2015) show LSFM as a tool capable of imaging 3D single cells in their ECM matrix. This demonstrates great potential for time-dependent traction force microscopy experiments and ECM degradation dynamics, two important aspects in studying the migration of cells.

## Integrated Light-Sheet Microscopy and Microdevices

The previous section has been mostly dedicated to custom LSFM setups that have attempted to accommodate standard sample mounting in wells, coverslips/slides, and PDMS microfluidic devices for cellular imaging. In this section the setups discussed are based on combining microdevice fabrication and optical hardware, requiring a certain degree of multidisciplinary knowledge.

### Reflected Light-Sheet Microscopy

Reflected light-sheet microscopy (RSLM) refers to a setup in which a reflective microfabricated surface is used to reflect the light-sheet orthogonally into the sample. This configuration should not be confused with reflective diSPIM method (Wu et al., 2017) which has been covered in Section “Inverted SPIM Configurations.” In RSLM (Gebhardt et al., 2013), or RLSM, an objective focuses a vertically aligned light-sheet into an atomic force microscopy cantilever that reflects it by 90° next to cells in a Petri dish. The fluorescence image is then detected by a second objective also in a vertical position (Figure 1G). By reflecting the light-sheet, the setup does not require two objectives placed at 90°. RSLM is shown to achieve single-molecule resolution in mammalian cells.

The main advantage of this setup is that it can be built over a commercial inverted microscope if the indicated changes are done on the hardware. A posterior study by Greiss et al. (2016) used commercially-available microprisms attached to the surface of a standard coverslip to reflect the light-sheet and perform single-molecule imaging in living *Drosophila* embryos. The use of microprisms suggests that, instead, micromirrors embedded in a microdevice could be used to perform RSLM on microdevices, a concept already developed and covered in the next section on single-objective SPIM.

### Single-Objective SPIM With Micromirrors

Single-objective SPIM (Galland et al., 2015), or soSPIM, operates in a similar fashion to RSLM. A microfabricated mirror in soSPIM substitutes the commercial microcantilevers and microprisms used in RSLM, but theoretically these could also be used to create the reflection for soSPIM. The use of micromirrors is more convenient because these can be produced from molds (Zagato et al., 2017) without a significant loss of quality in an effort to upscale toward inexpensive and disposable devices.

**Figure 3:**
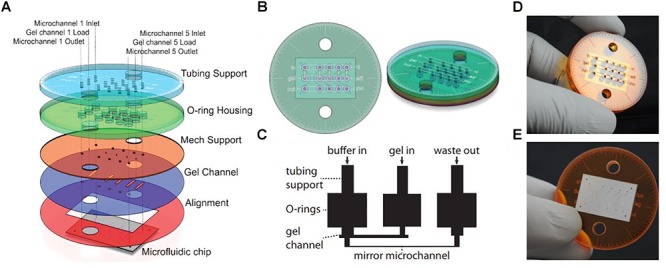
Single-objective LSFM microfluidic chip layout [Figure 6 from Meddens et al. (2016)]. **(A)** Exploded diagram of the different PMMA layers packaging the microfluidic chip. **(B)** Top and side view images of the assembled chip. **(C)** Schematic of the microfluidic device showing channel connections. **(D)** Photograph of the top of the microdevice. **(E)** Photograph of the bottom of the microdevice sealed with a coverslip.

An advantage of soSPIM is that only a single objective is used (Figure 1H) and can be installed into a standard inverted microscope if the appropriate hardware modifications are performed. Like RSLM, soSPIM allows for 3D single molecule resolution imaging of whole cells or cell aggregates, and the use of larger mirrors has demonstrated *Drosophila* embryo imaging. Another relevant aspect of soSPIM is that different research groups have successfully attempted to reproduce and improve it. For example, a full microfluidics device for high-speed whole-cell 3D super-resolution (Meddens et al., 2016) has been produced (Figure 3). The reflective surface forms the side wall of a microfluidic channel, microfabricated from a silicon wafer and sealed with a bonded glass coverslip. The chip is packaged in a case constructed from layers of laser-cut PMMA (polymethylmethacrylate). Meddens et al. (2016) take images of single cells to show an increase in imaging speed, improved contrast and deceased photobleaching when compared to an epi-fluorescent microscope.

**Figure 4:**
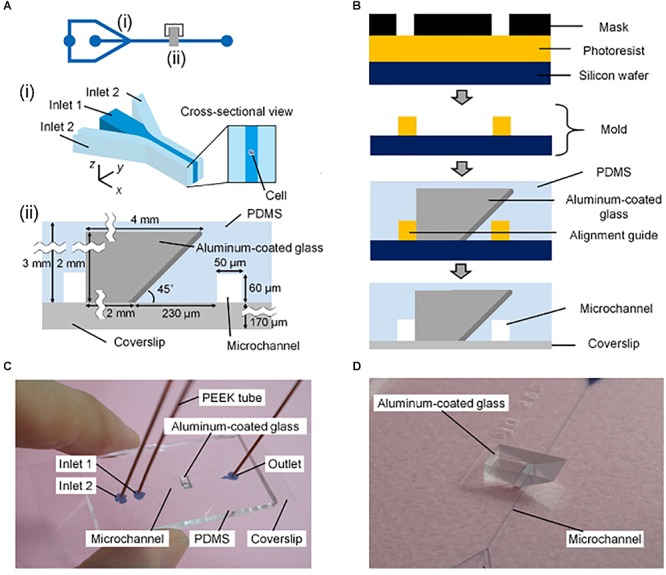
Mirror-embedded microfluidic chip for single-objective plane illumination [Figure 3 from Miura et al. (2018)]. **(A)** Schematic of the channels in the microfluidic device. (i) Hydrodynamic focusing region. (ii) Optical interrogation region. **(B)** Diagram summarizing the microfabrication process. **(C)** Photograph of the microfluidic chip. **(D)** Close-up photograph of the microchannel and aluminum-coated reflective glass.

Single-objective SPIM with micromirrors has also been adapted to flow cytometry (Miura et al., 2018). In this case, a mirror-embedded PDMS microfluidic chip (Figure 4) hydrodynamically focuses cells to obtain improved-contrast images at flowing speeds comparable to those of conventional non-imaging flow cytometers. The method was validated by classifying large populations of microalgal cells and human cancer cells, opening the door to high-throughput single-cell analysis.

### On-Chip Light-Sheet Microscopy

**Figure 5:**
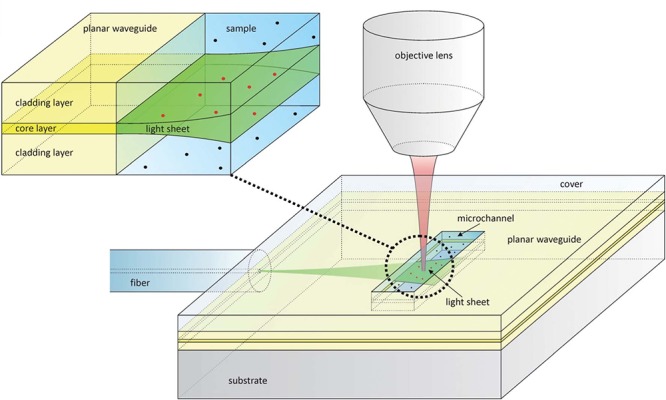
Design of a microfluidic chip with integrated waveguide for on-chip light-sheet illumination [Figure 1 from Deschout et al. (2014)]. Laser light (green) from a fiber optic enters the planar waveguide and is confined in the vertical direction, but spreads horizontally forming a sheet of light that exits in the microchannel. The fluorescence light (red) is collected by an objective lens. The red dots show fluorophores that are excited by the light-sheet, while black dots are those that are not. Only the fluorophore molecules traversed by the light-sheet are excited, removing out of focus noise.

**Figure 6:**
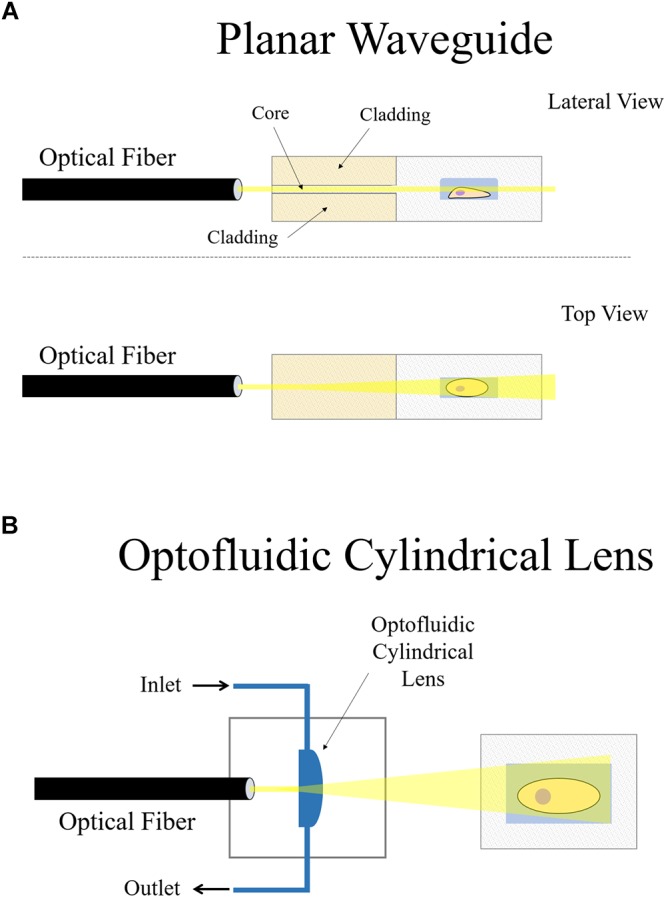
On-chip plane illumination methods based on optical fiber delivery: **(A)** A microfabricated planar waveguide allows the light from the optical fiber to expand laterally but not vertical. Confining the light in such a way creates an illumination plane which emerges at the microchannel where the sample is placed. **(B)** In this case, an optofluidic cylindrical lens creates a plane of light from an incident beam. The focus of such cylindrical lens can be changed if liquids of different refractive indices are employed. Two microchannels connect to the cylindrical microfabricated gap to change the fluid.

An example of on-chip LSFM has been covered in the previous section on soSPIM (Meddens et al., 2016; Miura et al., 2018) where micromirrors embedded in PDMS microfluidic devices are used for imaging or flow cytometry experiments. However, there are other experiments that have integrated microdevices and LSFM to, for example, diagnose size and concentration of membrane vesicles in biofluids (Deschout et al., 2014). In this paper the authors present an inexpensive disposable microfluidic chip for on-chip light-sheet illumination. Laser light enters a planar waveguide from an optical fiber, confining light in the vertical direction but spreading it horizontally (Figures 5, 6A). A light-sheet emerges at the microchannel where the sample is located. Light-sheet illumination improves the contrast of the fluorescence signal since out of focus fluorophores remain in the dark. The device is disposable, which is preferred to avoid extensive cleaning procedures and sample contamination. The group demonstrates light-sheet illumination in a mass-manufacturable chip close to that achieved on standard LSFM systems, substantially increasing the information provided by traditional epi-fluorescence illumination.

Another interesting approach that makes use of an optical fiber in a microdevice to create a plane of light was designed by Paiè et al. (2016). Femtosecond laser micromachining was used to create a microdevice for continuous flow imaging (Figure 6A), 3D reconstruction and high-throughput analysis of large multicellular spheroids at subcellular resolution. In their setup, optofluidic cylindrical lens are used to create the plane of light from an incident beam coming from an optical fiber (Figure 6B). The sample is then circulated by a microfluidic pump such that it traverses the light-sheet at a constant flow rate for imaging. The chip is a very small device that requires no external moving parts nor manual positioning of the sample. It can be easily mounted on both conventional wide-field or SPIM microscopes, demonstrating another feasible arrangement of how LSFM can be combined with microdevices.

### Custom Microdevices for Light-Sheet Microscopy

One of the greatest advantages of microdevices is customization, which allows them to be uniquely designed for specific applications, within the restrictions and limitations that come with microfabrication of photolithographic molds and soft lithography. In this section, examples of microfluidic devices that have been built for already-existing LSFM hardware or that require minimal modifications will be covered. Note that some on-chip approaches previously discussed (Deschout et al., 2014; Meddens et al., 2016; Paiè et al., 2016; Miura et al., 2018) make use of a light-sheet traversing microchannels for imaging of cells or single molecules. A particular distinction will be made between microfluidic devices in capillaries and those made from PDMS because of a difference in material properties, research interest and fabrication, acknowledging that PDMS-based microdevices have emerged as their own field of research.

#### Microfluidics in Capillaries

Some LSFM designs for small-sample analysis include the use of glass capillaries. For example, if using a traditional SPIM setup, single-cell resolution can be achieved by growing cells in collagen and then embedding the collagen in agarose. A glass capillary can be used to hold the sample, which is scanned with a light-sheet (Swoger et al., 2014).

A more elaborate setup shown previously (Miura et al., 2018) made use of flow-controlled conditions to perform the scan itself, removing the need of ETL or motorized stages. Similarly, glass capillaries have been used in flow cytometry for classification of phytoplankton (Wu J. et al., 2013) and identification of senescent cells (Lin et al., 2018). These devices hydrodynamically focus the cells in a square flow capillary. The cells within the capillary traverse an excitation light-sheet and the fluorescence image is recorded. In these setups, flow rates must be carefully balanced to maximize axial resolution, throughput and sensitivity.

Flow-driven sectioning can achieve higher throughput for a variety of reasons; straightforwardly, because the sample does not remain static during 3D imaging (Wu J. et al., 2013). Also, the use of LSFM in flow cytometry provides additional morphological information which is essential to some applications. This has allowed image processing and machine learning algorithms to perform automatic classification between different types of human cells (Lin et al., 2018) with potential applications in clinical diagnosis. In these studies, the square shape is important to avoid distortion of the light-sheet when it penetrates perpendicularly into the capillary. Squared glass microcapillaries have been previously used for LSFM of 3D cell cultures (Bruns et al., 2012) where they are applied to image a cell spheroid within a microcapillary. Here, the microfluidic flow is used to renew the culture medium and not to drive image sectioning. The authors explain the limitations of the geometry: the illumination plane suffers distortions at the edges of the capillary where the glass is not exactly perpendicular to the light-sheet, so imaging is restricted near the edges of the channel. For the flow cytometry applications discussed previously, shining the light-sheet close to the edges of the capillary is avoided by hydrodynamically focusing the cells toward the center of the channel. Still, this geometrical issue – which also occurs in PDMS microfluidic devices – can be solved by constructing the channel from a material with the same refractive index as the culture medium, as will be seen in the upcoming example.

In an effort toward systems for massive drug screening, a FEP tube (Kaufmann et al., 2012) was used as a sample holder for automatized high-throughput LSFM imaging of zebrafish and three-dimensional cancer cell cultures (Gualda et al., 2015). The use of FEP is advantageous because it has a refractive index close to that of water. The distortion of the light-sheet arising from the interface between materials of different refractive indices is significantly reduced if the tube is surrounded by water and water-immersion objectives are used. This has enabled to image the sample inside the tube without a detrimental effect on the optical quality, overcoming the geometrical problems posed by Bruns et al. (2012) when a using a glass capillary as a sample holder.

The properties of fluorinated ethylene polymers such as FEP or Teflon as materials for microfluidic chips, especially compared against PDMS, has been studied and documented (Ren et al., 2011). The refractive indices of materials like Teflon (*n* = 1.35–1.38) and FEP (*n* = 1.344) are very close to those of water (*n* = 1.33) and culture medium (*n* = 1.33–1.38). This could resolve many issues regarding the quality of optical interfaces between the objective(s) and the sample, which is one of the main translational drawbacks between microdevices designed for inverted or confocal microscopes and fluorescence light-sheet microscopes.

#### Microfluidic Devices From PDMS

Most microdevices for cell imaging are used in brightfield and widefield microscopy, and may also be used with confocal microscopy due to the single optical axis employed. Although LSFM requires two optical axes for emission and excitation, some microdevices can also be used with LSFM if the microscopy setup is built for standard sample mounting, as previously discussed, or if some steps are taken to devise another viable optical axis on the side of the microdevice.

Following on the applications of LSFM for flow cytometry, a simple single-channel PDMS microfluidic chip was used for high-throughput cell counting and imaging of yeast cells (Regmi et al., 2013). The light-sheet is able to cover the entire channel omitting the necessity of flow focusing and point-scanning. Fluorescently-labeled yeast cells were successfully imaged at low flow rates with improved count rate compared to existing systems. A low flow rate is important to prevent rupturing of the cells from shear stress against the channel walls; an alternative would be to hydrodynamically focus the cells at the expense of reducing the interrogation area. Cylindrical lenses of varying focal lengths can be changed in this setup to create light-sheets of varying dimensions. Posteriorly, the group implemented a tilted light-sheet under very low flow rates for automatic cell counting. To this end, image processing algorithms were developed for LSFM images of sub-cellular resolution. Cell counting and simultaneous visualization of fluorescently-labeled mitochondrial network in HeLa cells during flow was reported (Regmi et al., 2014).

**Figure 7:**
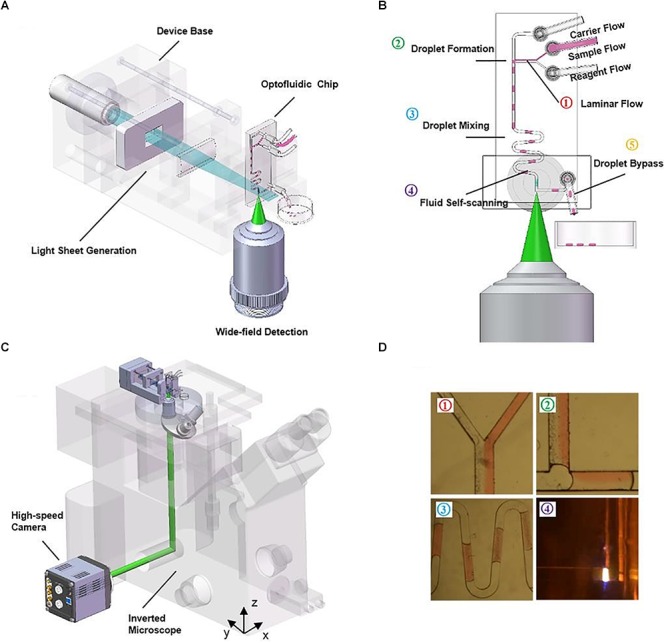
Compact, rapid, and cost-effective optofluidic device capable of preparing customized sample droplets [Figure 1 from Jiang et al. (2017)]. **(A)** Diagram of the device structure that includes the illumination optics, a microfluidic chip and a 3D-printed base. **(B)** The working scheme of the flow-based light-sheet scanning of droplets. The functions of droplet formation, mixing, LSFM imaging and bypassing are sequentially integrated, from upstream to downstream. Imaging is performed by the microscope objective from the side facet of the microfluidic device. **(C)** The device can be mounted on an inverted microscope for sustained image acquisition. Flow-based scanning eliminates the need of motorized stage and increases throughput. **(D)** The pictures of the actual droplets traveling through the microchannels.

The microfluidic devices shown in these publications (Regmi et al., 2013, 2014) are fairly simple, with a single straight channel connecting the inlet and the outlet. More advanced PDMS microdevices have also benefitted from the use of LSFM. Jiang et al. (2017) developed a compact, portable, rapid, and cost-effective optofluidic device capable of preparing customized sample droplets (Figure 7). Three syringe pumps containing oil (carrier flow), fluorescent microparticles in solution (sample flow) and a diluting solution (reagent flow) control droplet size, microparticle concentration, and flow speed. Flow elements such as T-junction, zigzag channels, and right-angled channels (Figure 7D) are used for creating the droplets, mixing the solutions, and withdrawing the sample from the scanning region, respectively.

These droplets flow through the device into a scanning region where they are imaged in 3D by LSFM. As seen before, flow-driven sectioning eliminates the need for mechanical scan. The chip offers high-throughput sample compartmentalization, manipulation, and volume imaging of microparticles, suggesting potential lab-on-a-chip applications such as embryo sorting and cell growth assays. Jiang et al. (2017) also comment on the irregular air-to-PDMS high-scattering interface that arises when cutting out the PDMS microdevice with a blade. In their protocol, an additional processing step is required to produce optically-flat facets that allow clearer imaging through that side of the chip. Some earlier-described LSFM setups for standard sample mounting, such as inverted SPIM configurations, could also be considered as a possible solution to high-scattering side interfaces.

Another microfluidic device of higher complexity has been designed for manipulating *C. elegans* specimens and delivering stimuli to monitor neural activity under widefield and brightfield illumination (Chronis et al., 2007). This microdevice was later translated from 2D to 3D imaging through the use of a custom-built SPIM (Rieckher, 2013). The setup is described as simple and cost-effective and able to perform SPIM and optical projection tomography (OPT) for fast 3D imaging of live *C. elegans* at single-cell resolution. An is objective placed on the standard optical axis directly facing the microdevice while the light-sheet is illuminated at 90° from the bottom through a refractive index matching fluid bath to avoid its distortion.

Apart from imaging and manipulation of *C. elegans*, single cells and droplets, microfluidic devices can be used for culturing. An interesting microfluidic device for cell spheroid culture and analysis (Patra et al., 2013) has a structure where a number of chambers located underneath a fluid channel can host uniform-sized spheroids. The cells to be deposited in the squared microchambers could be harvested from a flow cytometer. The authors also propose a device fabricated such that the cultured spheroids can be imaged with SPIM, having a vertical and a horizontal objective. Cuboidal chambers may be arranged in a pattern along several oblique parallel lines so that each chamber can be selectively illuminated with the light-sheet at a time to prevent overlap of the excitation and/or of the signal.

## Discussion and Outlook

The combination of light-sheet fluorescence microscopy and microfluidic devices has shown, so far, three ways of combining both techniques:

(1) Light-sheet setups for imaging in microdevices and standard sample holders.
(2) Microdevices designed and adapted to existing LSFM imaging stations.
(3) Microdevices that have built-in or are integrated with light-sheet imaging.

In great part, these fields of engineering rely strongly on custom-made systems that must be reproducible. Some designs have been successfully reported by different groups and used in meaningful and novel biology experiments, while others offer a proof-of-concept of the hardware that has been built. The most relevant design characteristics that have facilitated the combination of light-sheet illumination and microdevice technologies are highlighted in what follows, from both the viewpoint of the optical setup, and from that of microdevice fabrication.

### Selecting the Light-Sheet Imaging Workstation

Optical hardware research groups that have approached LSFM have created a variety of systems that offer different characteristics. Since a fluorescence imaging workstation can be a large investment for cell biology laboratories, selecting its appropriate benefits becomes paramount. Here, some of the capabilities that a system should offer for single-cell imaging and subcellular resolution are discussed.

Related to sample sectioning, the use of ETLs over mechanical motion is preferred. ETLs are a piece of optical equipment that can adjust their focus according to the current being applied, within a range limit. ETLs have very a fast response and allow for more compact setups with less mechanics. Additionally, the cost of an ETL is lower than that of a microstep piezoelectric stage. Motor stages would still be required to place the sample in position, but the precision of such movements becomes particularly essential when these are driving the scan. There are several benefits associated to tunable lens over mechanical stages. First, ETLs offer increased scan speeds (Fahrbach et al., 2013) and therefore, an increase in temporal resolution which is crucial when studying dynamic processes in cell biology. As shown in Table 1, not all methods can or have included tunable lenses in their setups, but they might offer other advantages which may suit a particular application.

Apart from temporal resolution, image quality and spatial resolution can be improved because the sample and the optical equipment remain static during sectioning (see Mickoleit et al., 2014 for an example where ETLs are applied to the beating zebrafish heart). Thus, the chance of blurring and misalignments decreases. Inertial forces during fast translations of a motor are avoided, which may unpredictably affect the adherence of the cells to the substrate or the flow of a fluid in a microchannel. There can also be space restrictions and limitations regarding sample mounting when the sample does not remain static and is translated back and forth from the detection objective for sectioning. This requires enough space between the objective and the sample to avoid collisions.

With respect to geometries, configurations that require a single objective have the advantage of having a single optical axis which facilitates their use in a standard microscope setup. Another advantage is provided by the systems that can take multiple views of the same scan, since it allows for a more homogeneous illumination of the sample while reducing shadow artifacts created by highly-absorbing/scattering regions in the images. Traditional SPIM accomplishes this by implementing a rotating stage that may acquire 360° views. In the case of samples that remain static or are linearly scanned, an extra set of cylindrical lens in SPIM together with a mirror galvanometer motor can offer multidirectional views (mSPIM) by creating a pivoting light-sheet (Huisken and Stainier, 2007). This type of pivoting has minor effects on the scan speed and in some cases may significantly reduce shadow artifacts. Multiple views can also be acquired through systems with more than one illumination objective such as diSPIM and multi-view LSFM (Krzic et al., 2012).

Regarding the imaging applications, most light-sheet systems covered above are suitable to image samples that range in size from single cells to small organisms. Nevertheless, there are two exceptions: single-objective SPIM is restricted to sample sizes no larger than small cell cultures, while reflected LSFM can image all the way from small organisms down to sub-cellular structures and single molecules.

### Designing the Microdevices

For any microdevice, it is advisable to use materials along the optical path with homogeneous refractive indices and with the interfaces perpendicular to the path of light, avoiding irregular (non-planar) flow elements such as grooves, and circular pillars, barriers, or chambers. The number of material interfaces should be kept minimal within the path of light because of the reflection and refraction effects. Also, when cutting PDMS microdevices the interfaces remain highly irregular. These interfaces are sometimes necessary for LSFM, since it requires two optical axes at 90°. To combat outer-surface irregularities which interfere with the light-sheet, an additional step is required to either flatten the surface or couple the refractive indices with a refraction medium bath. Alternatively, Teflon and FEP – which have refractive indices close to those of water – can be used in building the microdevice. The main drawback of this non-mainstream fabrication technique is that it is not as straightforward as soft lithography with PDMS.

Another factor to keep in mind is accuracy and reproducibility in microfabrication. This can be especially critical for those systems in which the optical performance directly depends on microfabricated elements, most noticeably: RSLM, soSPIM, and on-chip LSFM. In all cases, the appropriate quality of microfabrication will be determined in great part by the desired application. For example, quantitative imaging of certain subcellular structures with RSLM or soSPIM will demand micromirror surfaces that are smooth and uniform. The use of irregular or imperfect molds – particularly in the aforementioned techniques – could require constant adjustment and/or recalibration of the optics leading to lower throughput and increased labor costs.

Finally, keeping the thickness of the microdevice as fine as possible facilitates sample mounting when objectives of higher numerical aperture and magnification are used; these are prevalent in subcellular resolution imaging and have shorter working distances with which to operate. This is important in PDMS microfluidic devices, where the height of the PDMS gel can be determined when pouring it in the mold.

## Conclusion

The review has mostly focused on light-sheet cell-resolution imaging in microdevices, going through several setups that have successfully accomplished it. Other applications of LSFM in microfluidic devices, such as flow cytometry, are also detailed. The temporal and spatial resolution render LSFM ideal for the study of dynamic processes in cell biology, and microdevices can offer a finely-controlled platform for experimentation. Thus, the combination of both emerging technologies is expected to grow, contributing to promising results in the area of cell motility and tracking; especially in the studying of essential processes like development, neurology, immune system, tissue repair, and tumor metastasis, all of which are of strong biomedical significance.

## Author Contributions

IA-S wrote the manuscript together with AM-V, supervised by JR, AM-B, JJV and MD.

## Conflict of Interest Statement

The authors declare that the research was conducted in the absence of any commercial or financial relationships that could be construed as a potential conflict of interest.
